# Which Doctor to Trust: A Recommender System for Identifying the Right Doctors

**DOI:** 10.2196/jmir.6015

**Published:** 2016-07-07

**Authors:** Li Guo, Bo Jin, Cuili Yao, Haoyu Yang, Degen Huang, Fei Wang

**Affiliations:** ^1^ School of Computer Science and Technology Dalian University of Technology Dalian China; ^2^ School of Innovation and Entrepreneurship Dalian University of Technology Dalian China; ^3^ Department of Healthcare Policy and Research Weill Cornell Medical College Cornell University New York City, NY United States

**Keywords:** recommender systems, feature selection, rank aggregation, key opinion leaders

## Abstract

**Background:**

Key opinion leaders (KOLs) are people who can influence public opinion on a certain subject matter. In the field of medical and health informatics, it is critical to identify KOLs on various disease conditions. However, there have been very few studies on this topic.

**Objective:**

We aimed to develop a recommender system for identifying KOLs for any specific disease with health care data mining.

**Methods:**

We exploited an unsupervised aggregation approach for integrating various ranking features to identify doctors who have the potential to be KOLs on a range of diseases. We introduce the design, implementation, and deployment details of the recommender system. This system collects the professional footprints of doctors, such as papers in scientific journals, presentation activities, patient advocacy, and media exposure, and uses them as ranking features to identify KOLs.

**Results:**

We collected the information of 2,381,750 doctors in China from 3,657,797 medical journal papers they published, together with their profiles, academic publications, and funding. The empirical results demonstrated that our system outperformed several benchmark systems by a significant margin. Moreover, we conducted a case study in a real-world system to verify the applicability of our proposed method.

**Conclusions:**

Our results show that doctors’ profiles and their academic publications are key data sources for identifying KOLs in the field of medical and health informatics. Moreover, we deployed the recommender system and applied the data service to a recommender system of the China-based Internet technology company NetEase. Patients can obtain authority ranking lists of doctors with this system on any given disease.

## Introduction

In the field of medical and health informatics, key opinion leaders (KOLs) are the doctors who can influence public opinion and lead the medical community through their research papers and clinic practices. These KOLs play important roles in the health care industry at every stage of their product life cycle. Therefore, there is a critical need for intelligent KOL identification services. Traditionally, consulting companies provided services for identifying KOLs by conducting user surveys. These business solutions use only a limited number of information resources and focus on a small number of involved clients. Advances in informatics technologies have enabled us to collect large amounts of medical-related data [1], which in turn provide a new carrier for KOL identification. To this end, we conducted a large-scale quantitative analysis of multisource medical-related data and developed a recommender system for effectively identifying KOLs of any given type of disease by using such data.

KOL identification is also important to patients, since KOLs can influence which doctors patients want to approach. Several websites provide information on relevant doctors for patients, such as Yelp and Zocdoc. Yelp provides user reviews of doctors, but the quality of the reviews is not guaranteed. Zocdoc works primarily as a front end for managing a doctor’s practice. The information used in both websites about doctors is relatively simple and not trustworthy.

In practice, one way to identify reliable KOLs is through referrals—in other words, the number of times a doctor is referred by another doctor. This can be treated as one type of social trust for doctors. In our method, we exploited coauthorship relationships and citation relationships to mimic such referrals. This process can be viewed as constructing doctor-centered networks from coauthorships and citations, which has been rarely studied (although there has been research on a patient-centered network [2]). On the other hand, although we cannot recognize good doctors only by counting their publications and all their citations [3], doctors whose papers are highly cited or who have published many papers in high-impact journals can promote their ideas and opinions to others more easily [4]. This is the same logic as that behind the PageRank algorithm for the Google search engine, which has also been used in the analysis of social network influence. In health informatics, KOL identification should encode objective and validated measurements of KOL activities, including academic publications, invited talks, quality of clinical research, patient evaluations, and media exposures. These activities should also be used as ranking features to identify KOLs.

The aim of this study was to develop a recommender system for identifying KOLs for any specific disease. Here we introduce the design, implementation, and deployment details of such a KOL identification system. Our system consists of 5 components: acquirement, integration, storage and access, modeling, and recommendation. The system is extensible and configurable, and has been deployed online for several months. In the recommendation component, we chose the profile of doctors, the expertise of doctors, and the social trust of doctors as the ranking features. The ranking function designed for KOL identification was constructed based on those features. We further developed an unsupervised ranking aggregation approach for KOL ranking. In a real-world deployment of our system, we also incorporated some external knowledge and optimized the settings of our system manually according to the recommendations of our operation team.

### Prior Work

KOLs are respected individuals who have a huge impact on other people’s opinions, actions, and behaviors in a given social network [5]. Nowadays, people seek opinions and advice for supporting various decisions (eg, regarding medical treatment) from KOLs. Therefore, the key question is how to effectively and efficiently identify KOLs [6].

For academic research, there are mainly two categories of methods for identifying KOLs. The first category uses primary data, such as self-designation and peer identification [7]. The second uses secondary data, such as publications and social networks [8]. Primary data are more difficult to collect but are more accurate and effective [9]. There are also some combined methodologies using both primary and secondary data [5].

The number of business solutions encouraging KOL identification in the health care industry has also been increasing. For example, Thought Leader Select offers KOL identification, profiling, engagement planning, mapping, interviews, and surveys services to over two dozen of the world’s largest biopharmaceutical and health care companies [10]. Moreover, a health care startup, HealthTap, constructed a doctor social graph to launch a service that maps doctors’ connections [11]. Their graph, called DOConnect, has 25 million doctor referrals and was generated with big data technologies.

### System Overview

**Figure 1:**
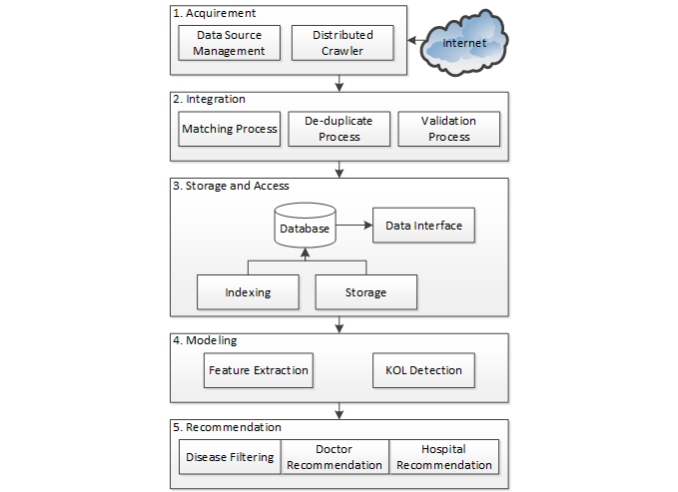
Architectural overview of the key opinion leader (KOL) identification system.

Figure 1 shows the architecture and workflow of our system, which consists of *acquirement*, *integration*, *storage and access*, *modeling* and *recommendation* stages.

#### Acquirement Stage

This stage focuses on acquiring health care information from the Internet automatically. We developed an advanced Web crawler [12] for collecting the doctors’ profiles and publications from multiple open data sources, which can be managed by rule-based operations.

#### Integration Stage

This stage aims to integrate the doctors’ profiles and publications through a data matching process. These data are further processed through a de-duplication and validation processes to improve their quality.

#### Storage and Access Stage

This stage provides the capability of storing and indexing the integrated data. Specifically, we used MySQL for database storage and indexing, and provided a data access interface via Web service application programming interfaces.

#### KOL Identification Stage

This stage identifies KOLs. In our system, this task is treated as a classic information retrieval task. Specifically, we used an unsupervised aggregation approach to integrate the ranking features of health care data for KOL identification.

#### Recommendation Stage

This stage provides several recommendation services based on the results of KOL identification. Specifically, the system can return the ranked KOL list and corresponding hospital list as recommendations for users based on their personalized specifications, such as disease category. The recommendation results can be further filtered with the detailed disease names.

## Methods

### Design and Deployment

In this section, we discuss the design and deployment of our KOL identification system in detail. This system is based on a previously published study [13].

#### Data Acquisition

To build our system, we used a Web crawler to collect large-scale health care-related data from multiple sources, including government public data, official hospital websites, professional health care websites, and medical companies’ information systems.

**Figure 2:**
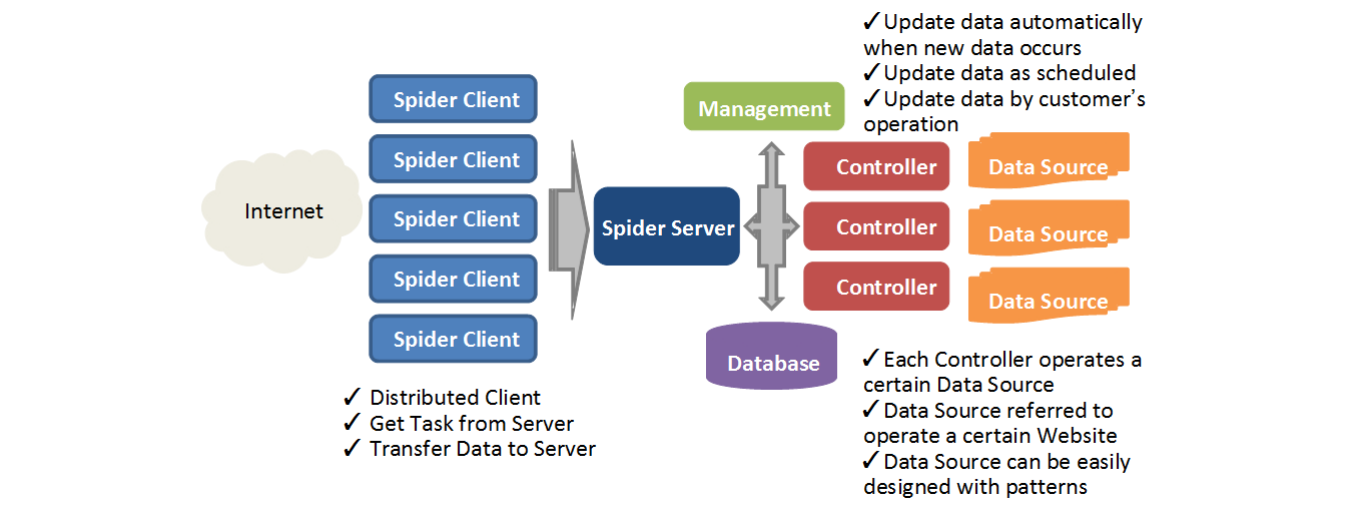
Data acquisition in key opinion leader identification system.

A Web crawler is usually set in advance for a specific website design, and thus it is difficult to modify the crawler when the target site is changed. To meet the system requirement of multiple-source data acquisition, it is necessary to redesign the Web crawler. Here we present an advanced method for implementing Web crawler task management based on Jun [12], which Figure 2 shows. This method has the following steps: (1) initializing the link address of a webpage to be crawled by the client, (2) packaging the link address of the webpage to be crawled into a task request to the server by the client, (3) sending an HTTP request from the server to the webpage to be crawled and returning the information required to the client, (4) receiving the information and processing the information on the client, (5) repeating the process and completing the webpage crawling in a crawling list sequentially. The proposed method provides a universal crawling framework for crawling different Internet content. In this way, crawlers for a special webpage can be quickly compiled, and thus the development can be much easier and more efficient. Furthermore, as the method is established based on the distributed Internet crawler framework, crawling efficiency can be further improved.

**Figure 3:**
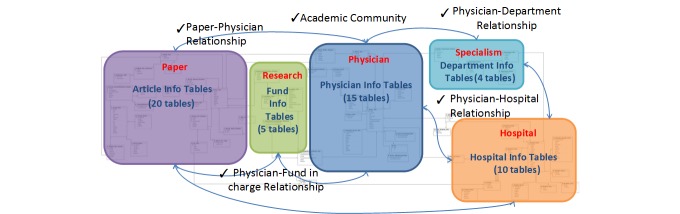
Database structure and information in the tables for data acquired by the key opinion leader identification system.

We also created a database to store the acquired data, which includes 54 tables (Figure 3). The structure of our database is extensible, and thus the database has the capability to incorporate more datasets in the future.

#### Data Processing

**Figure 4:**
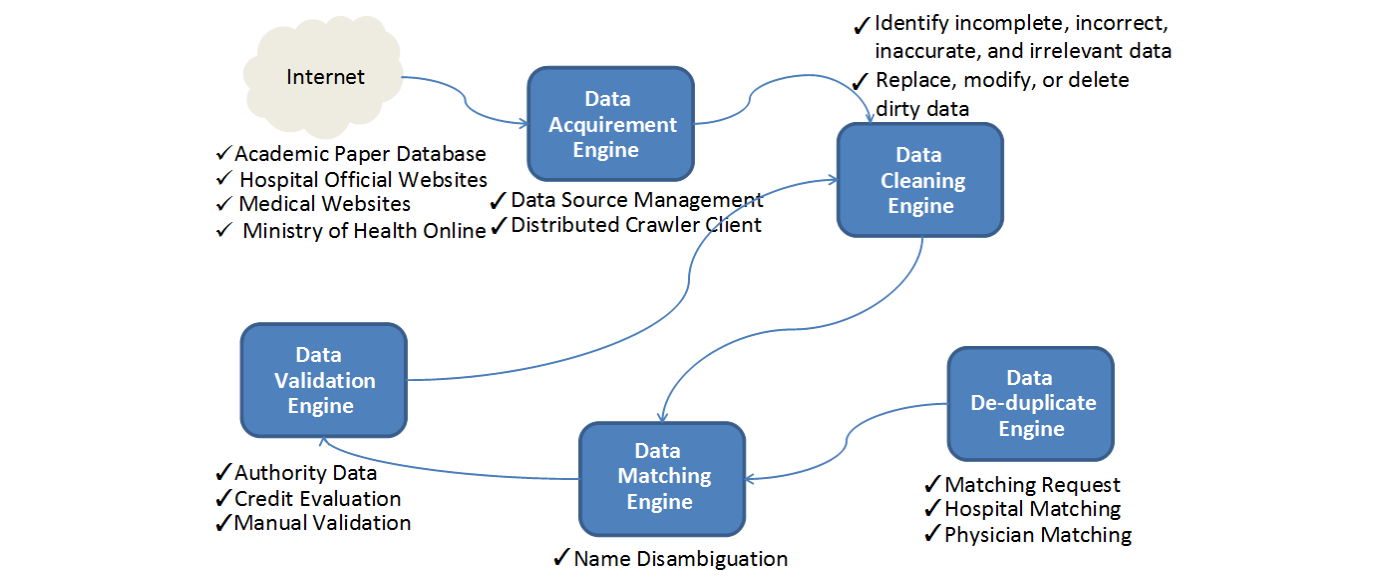
Data preprocessing workflow in the key opinion leader identification system.

As Figure 4 shows, our system processes data in the following 4 steps. The first step is to clean the acquired data. Since there is a lot of noise in the original data, we first identify the incomplete, incorrect, inaccurate, and irrelevant parts. Then, we clean, replace, modify, or delete such “dirty” data.

The second step is to match the multisource health care information. Since a hospital would have several names with different acronyms, the hospital names are matched using alias lists. Actually, the process of merging multisource information encounters a lot of name errors. The names of doctors are matched using Chinese pinyin (romanized Chinese ideograms), which can reduce written errors in Chinese characters.

The third step is to de-duplicate the doctors, since many names are duplicated. Therefore, we consider the same name appearing in the same hospital with the same specialty to be a single doctor, so that we can reduce the number of duplicated names.

The final step is to validate the multisource doctor data. In particular, we validate the information’s consistency across multiple sources. For any specific doctor, we retain her or his information from more reliable and more recent sources and discard the information from other sources when inconsistency appears. We also apply a manual check as the last step.

In our system, we use only academic papers in the domain of medicine to identify KOLs. Because not all authors of a paper are doctors, we match the paper’s authors to the doctor dataset to identify the doctors more accurately.

#### Data Analysis

Our health care datasets contain almost all the registered doctors in China from the Chinese Ministry of Public Health. There are in total 2,381,750 doctors in the dataset. The profile of each doctor includes sex, age, specialty, title, employer, work experience, and resume. This information is collected from multiple sources. We have also crawled information for 106,021 hospitals in China. Hospitals are divided into 3 grades and 3 classes: grade III class A is the highest level, and grade I class C is the lowest level. Most doctors are employed in hospitals in grade II class A (41.5%) and grade III class A (31.7%).

In addition, our dataset contains information about all 1103 medical journals published in China. There are in total 3,657,797 papers (1980–2014) in the dataset. Information about each paper includes the journal name, publication date, volume, title, list of authors, authors’ affiliations, classification identification, abstract, keywords, and references. Based on this information, we constructed a coauthorship network among doctors. For example, if 2 doctors coauthor at least one paper, then there will be a cooperative relationship between them. An analysis found that most doctors have no more than 50 coauthors, while the largest number of coauthors was over 300.

#### Web App

**Figure 5:**
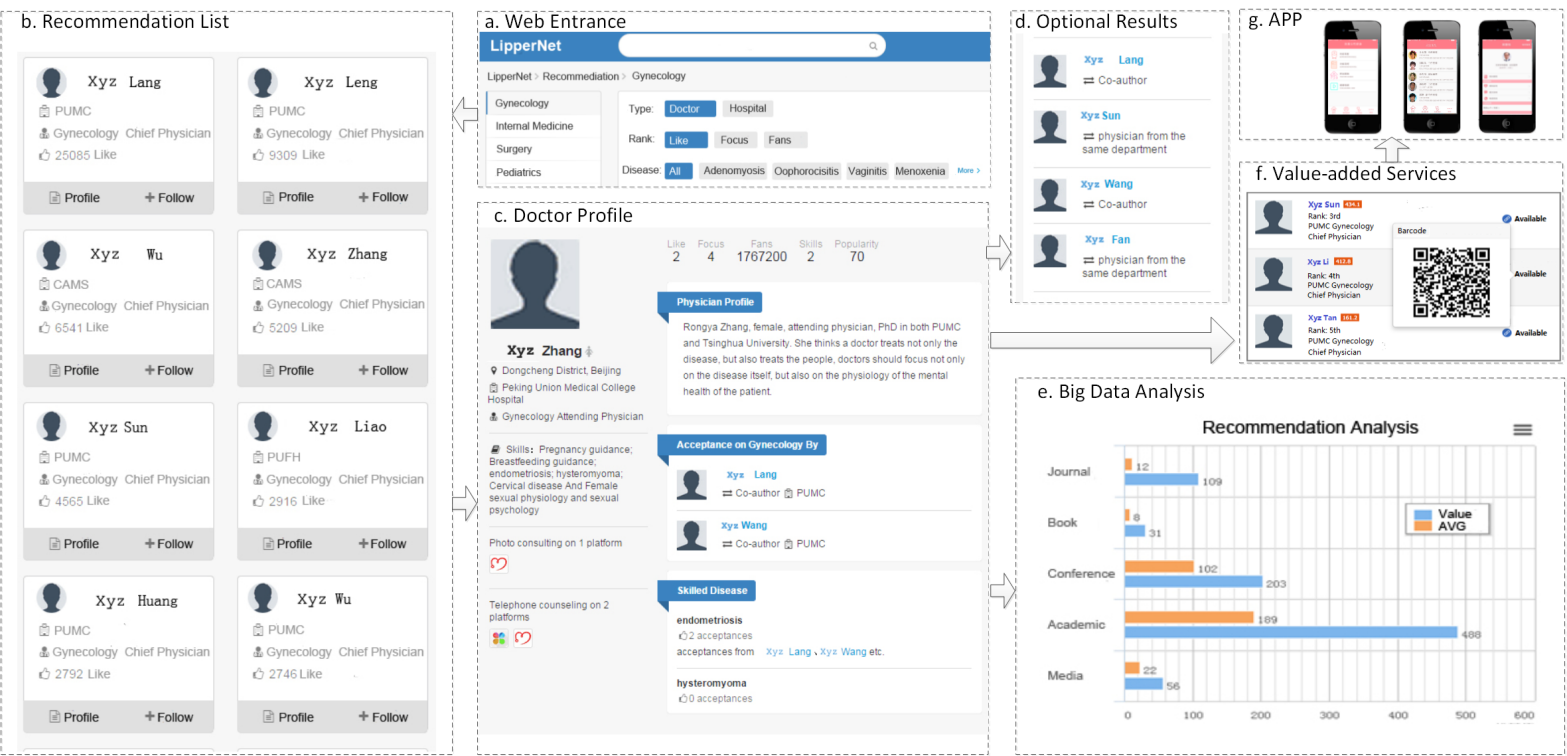
Screenshots from the Web app showing doctor recommendation and content analysis functions.

Our system can produce recommendations for pharmaceutical companies and patients, and its Web-based front end enables content analysis and recommendations for users. Figure 5 shows screenshots from the Web app and the steps in making doctor recommendations.

### KOL Identification

In this section, we introduce the technical details of our KOL identification approach. First, we formally defined the problem of KOL identification in this study. Given a disease category *c* as an element of the set *C* and a set of doctors *D*={ *d*_1_, *d*_2_, ..., *d*_n_}, the problem of KOL identification is to find the top *K* authoritative doctors in *D* for category *c*. Intuitively, this problem can be regarded as a classic information retrieval task, where the major challenge is how to define the ranking features for effectively linking doctors’ expertise and disease categories. In the following we introduce the detailed ranking features used in our system and how to integrate these features for KOL identification.

#### Ranking Features for KOL Identification

In our system, there are 3 types of ranking features for KOL identification, namely *doctor’s profile*, *doctor’s expertise*, and *social trust of the doctor*.

The doctor’s profile is the basic descriptive information in his or her resume, such as demographic information, academic background, and professional activities. The system extracts 5 features based on the doctor profiles in our datasets: *professional duration*, *academic title* (eg, Full Professor), *professional title* (eg, Physician), and the *hospital level* where she or he works (eg, grade III class A). Table 1 (top) describes these features.

**Table 1:** Description of ranking features in the key opinion leader identification system.

Feature type	Feature	Description
Profile features	Professional duration	Working years of the doctor
	Academic title	None, Assistant Professor, Associate Professor, Full Professor
	Professional title	None, Physician, Resident Physician, Physician in Charge, Associate Chief Physician, Chief Physician
	Hospital level	GI-A, GI-B, GI-C, GII-A, GII-B, GII-C, GIII-A, GIII-B, GIII-C^a^
Expertise features	Number of publications	Number of academic publications by a doctor in the given disease category
	Patient rating	Average rating of the doctor given by his or her patients
	Expertise label	Correspondence of the given disease category with a doctor’s expertise labels
Social trust features	Coauthorship	Evaluation of the degree of collaboration between doctors
	Publication citation	Evaluation of the doctor’s authority
	Social recognition	Evaluation of the degree of the doctor’s social recognition

^a^Grade and class of hospital (eg, grade I class A).

The doctor’s expertise is used to evaluate the expertise level of a doctor with respect to the given disease category. Specifically, we extract 3 expertise features, described in the middle part of Table 1. The first feature is the *number of publications* a doctor has in a given disease category. The second feature is the doctor’s average *patient rating* and can be used to evaluate his or her treatment in a given disease category. The third feature, *expertise label*, denotes the correspondence between a given disease category and a doctor’s expertise labels.

**Figure 6:**
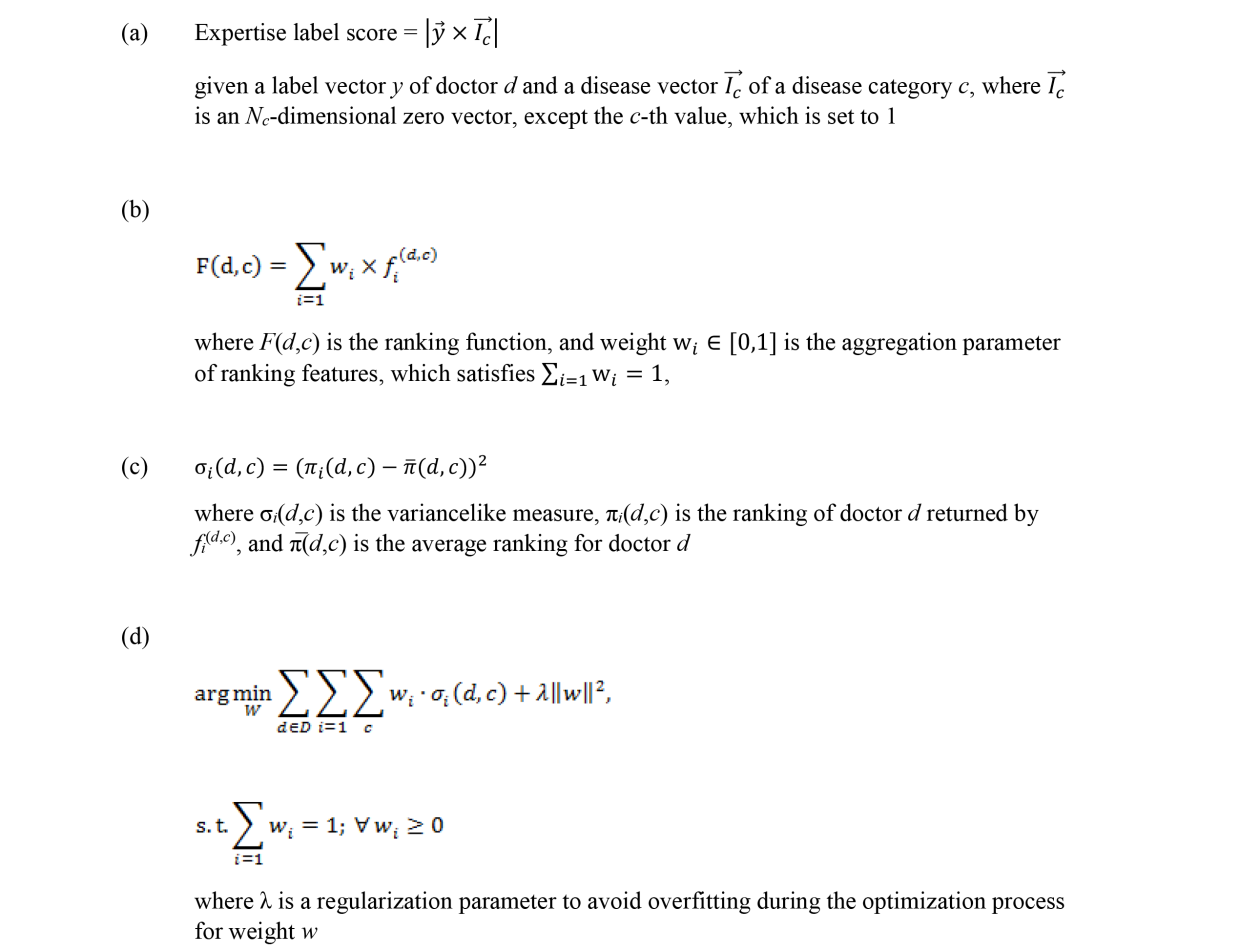
Equations used for the ranking functions.

To construct the expertise label feature, we label each doctor with a vector *y*. First, we select a group of doctors randomly and manually label each doctor with the disease category with which they are most experienced. Then we apply the label propagation algorithm [14] on multiple networks to predict labels corresponding to the expertise of all doctors in our datasets. After the labeling, we have *N*_d_ label vectors in total, where *N*_d_ denotes the number of doctors in the set of doctors *D*. Each label vector can be represented as an *N*_c_-dimensional vector, where *N*_c_ is the number of disease categories in *C*. Each dimension of the vector represents the extent to which a doctor is skilled in treating a specific disease category. If a doctor is perfect in treating a specific disease category, the corresponding value in the vector is set to 1; otherwise, if he or she is completely unable to treat the disease, the value is set to 0. Therefore, the expertise label score is computed as shown in equation (a) (Figure 6).

We evaluate the doctor’s social trust with respect to a given disease category, which can be very useful for identifying KOLs among doctors. Specifically, we exploit 3 authority scores as social trust features in our system. The first score is *coauthorship*, which is defined to evaluate the degree of collaboration between doctors. Specifically, given a doctor *d* and all of his or her publications *P* in the given disease category *c*, the coauthorship is represented by the number of different authors in publication *P* except *d*. Generally, the more partners the doctor has, the stronger the academic influence she or he has. The second score is the *publication citation*, which is computed as the number of publications *P* that doctor *d* published in the given disease category *c* that were cited. The publication citation is a good performance indicator of his or her academic authority. Third, we extract *social recognition* as a feature to support the judgment of whether a doctor can be trusted. Specifically, social recognition is indicated by the number of the doctor’s social fans. For example, the doctor’s social recognition score *S*_d_ is set to 2 if he or she has 20 social fans, the score is 3 for 100 social fans, and so on. However, not everyone has social networks, that is to say, not every doctor has social fans. If doctor *d* doesn’t have a social network, then social recognition is set to 0. The feature descriptions are detailed at the bottom part of Table 1.

#### Ranking Function for KOL Identification

After the above ranking features are constructed, the remaining task is how to integrate them for KOL identification. A common way is to define a linear ranking function with unknown feature weights as parameters, which are obtained from training data [15]. However, our data lack sufficient and reliable information that can be regarded as ground-truth ranking of doctors for each disease category, which makes it difficult to use a traditional supervised learning approach to obtain a ranking function. To solve this problem, in our system we use an unsupervised aggregation approach proposed by Zhu et al [16] for integrating ranking features.

Specifically, first we manually transform all categorical features into numerical values so that they can be used as scores for ranking doctors. For example, we transform the values of the feature *academic title* from none, Assistant Professor, Associate Professor, and Full Professor to 0, 1, 2, 3, and 4, respectively. Then, we implement normalization by subtracting the mean and dividing the standard deviation for all numerical features. After this, the ranking features of a given doctor-disease tuple (*d*, *c*) can be denoted as { *f*_1_^(d^^,^^c^^)^, *f*_2_^(d^^,^^c^^)^,..., *f*_m_^(d^^,^^c^^)^}, where *m* is the number of features we extracted. Meanwhile, the ranking function *F* (*d*, *c*), which indicates the expertise score of *d* in *c*, is defined by equation (b) (Figure 6). Given a set of doctors *D*, we select *n* ranked lists with feature scores. Then π_i_(*d*, *c*) is the ranking of doctor *d* returned by *f*_i_^(d^^,^^c^^)^, and π̅(*d*, *c*) is the average ranking for doctor *d*. Thus, for feature *f*_i_^(d^^,^^c^^)^, consistency is calculated by the variance-like measure in equation (c) (Figure 6). The smaller σ_i_(*d*, *c*) is, the larger the weight, and vice versa, of *f*_i_^(d^^,^^c^^)^ should be assigned. Thus, the feature aggregation problem is defined as an optimization problem as shown by equation (d) (Figure 6).

The above problem can be solved by a gradient-based approach [16]. After learning the feature weights, we can rank the doctors with different disease categories for KOL recommendation. Our algorithm is based on the algorithm developed by Zhu et al [16] and Wang et al [17], which aims at minimizing the global inconsistency (reflected by the variance of ranking results) of all ranking measures.

## Results

In this section, we present the empirical results for validating the effectiveness of our system in terms of KOL identification with all of the data we crawled.

### Experimental Data

As mentioned above, there are many doctors in our system (2,381,750 doctors), but only a small percentage of the doctors can be identified as KOLs. Most doctors are at low-level health organizations and we have little information for them. To evaluate our proposed method, we used a subset of our data as the experimental data, which we collected from We Doctor. This real-world data set includes 29,203 doctors in more than 7,000 expert teams all over China. Most of these doctors are experts in more than one discipline. Furthermore, each expert team has a leader, who can be treated as a KOL. That is to say, the leader of the expert team can influence at least the team members with his or her medicinal opinions.

First, we analyzed doctors’ profiles and discovered that more than half of the doctors (up to 63.07%, 18,418/29,203) in the experimental dataset have senior titles, such as Chief Physician and Associate Chief Physician. In contrast, 35.73% (141,745/396,718) of doctors have senior titles in our full dataset from the top category of hospitals (grade III class A). This indicates that we used a subset of doctors who were more likely to be experts. Second, by analyzing patients’ reviews, we found that most indicated the highest levels of satisfaction (ie, levels 8 and 9). A fairly large number of reviews reported dissatisfaction (ie, level 1). Few reviews indicated other levels of satisfaction. This indicates that patients tended to review doctors at the extremes, that is, either satisfied or dissatisfied, even for the experts. Third, an analysis of doctors’ social media followers showed that most doctors had few followers, although some “star” doctors had a large number of followers.

### Evaluation of KOL Identification

In China there is no public authority ranking list of doctors. Therefore, in this study, we evaluated the proposed approach with the doctor review and rating data gathered from our data service platform. We collected review logs of doctors and diseases entered into our data service between November 1, 2015 and January 31, 2016. There were 3496 review logs for 1133 doctors and 7823 review logs for 51 diseases.

**Figure 7:**
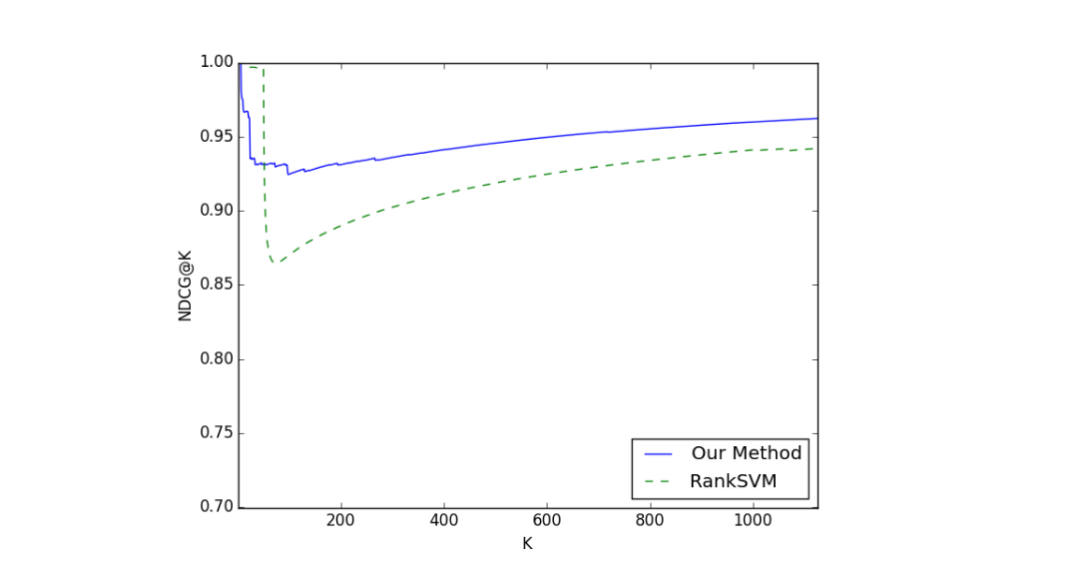
Evaluation by normalized discounted cumulative gain (NDCG) at cutoff rank K of recommendation performance by two approaches (RankSVM and the proposed method) based on data from November 1, 2015 to January 31, 2016.

We used RankSVM [18] as the baseline and used normalized discounted cumulative gain (NDCG) [19] to evaluate the performance of the recommendation result. NDCG indicates the ranking performance with a cutoff rank *K*. Figure 7 shows the recommendation performances of the two approaches. Our approach outperformed the baseline by a significant margin, especially for smaller *K* when *K* is larger than a threshold of 50.

We also did a focus group study with 1341 gynecologists in Beijing. To establish a reference standard, we invited 6 evaluators (3 faculty members with a medical background and 3 graduate students) to provide human judgments with scores of 4 (definite expertise), 3 (expertise), 2 (marginal expertise), 1 (little expertise), and 0 (no expertise). Group members based their judgments mainly on what they thought about the doctor’s professional activities and reputation. After this user evaluation, each doctor was assigned a judgment score. We averaged the judgment scores and used them to rank the doctors. We selected the top 30 doctors to build the ground truth. Then we implemented our system and other systems (Haodaifu, Beijing, China; and DXY, Hangzhou, China) with similar functions in the evaluation dataset. We used the precision at 10 documents retrieved, R-precision, and mean average precision as performance measures [20].

**Figure 8:**
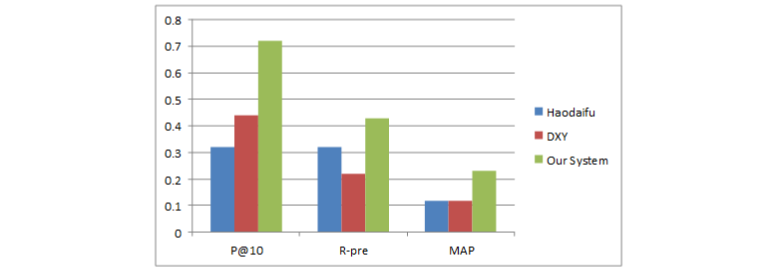
Recommendation performance of different approaches on a small data subset evaluated by precision at 10 documents retrieved (P@10), R-precision (R-pre), and mean average precision (MAP).

Figure 8 shows the results of KOL identification. The evaluation terms (precision at 10 documents retrieved, R-precision, and mean average precision) of different diseases were averaged to obtain the experimental results. Our method performed better than the others.

## Discussion

We investigated and proposed new data mining models for KOL identification. Moreover, we have developed and deployed the KOL identification system. Over the past year, we have been deploying and testing our system online. The following section describes a case study that we applied to our system to verify the applicability of our proposed method.

### Case Study

We selected 5 diseases (adenomyosis, ovarian cyst, vaginitis, menoxenia, and cervicitis) from common gynecological categories for a case study. Table 2 shows the top 5 recommendation results of gynecologists in Beijing. There were 1341 gynecologists, most of whom were leading doctors for all of China. Our results show a high degree of overlap. Adenomyosis and menoxenia have the same doctor in the first position, as do ovarian cyst and vaginitis. This suggests that a leading doctor is ranked reasonably higher in similar or associated diseases, such as ovarian cyst and vaginitis. In contrast, the results of adenomyosis and ovarian cyst are quite different for the two diseases, which have less similarity or association. We also found that most of the recommended doctors were committee members of the gynecology branch of the Chinese Medical Association. For example, Jinghe Lang was the chairman of the gynecology branch. This validates our recommendation results.

**Table 2:** A case study of key opinion leader recommendations.

Diseases	Doctors
Adenomyosis	Jinghe Lang, Jinhua Leng, Zhufeng Liu, Dawei Sun, Yingfang Zhou
Ovarian cyst	Zhaohui Liu, Fengzhi Feng, Bin Li, Jinsong Han
Vaginitis	Zhaohui Liu, Qinping Liao, Dai Zhang, Li Geng, Shuqing Jiang
Menoxenia	Jinghe Lang, Shan Deng, Ying Jin, Jian Shen, Ming Wu
Cervicitis	Qinping Liao, Li Geng, Lingying Wu, Wenhua Zhang

We successfully applied our recommender system data service to NetEase, which is a leading China-based Internet technology company and is listed on NASDAQ as NTES.

### Conclusions

The KOL identification system we have developed can provide better KOL identification for pharmaceutical companies and patients. Our system integrates profiles of doctors and academic publications in the domain of medical science. This paper introduces the design, implementation, and deployment of our system. Specifically, we first acquired health care data from multiple sources using a Web crawler. Then we integrated the data into one system and preprocessed them using matching, de-duplication, and validation processes. We designed a storage system for the processed dataset and performed some basic statistical analyses on the dataset. Further, we proposed an approach of unsupervised ranking aggregation. Finally, this system can make recommendations to pharmaceutical companies and patients based on the proposed methods.

## Abbreviations

KOL: key opinion leader
NDCG: normalized discounted cumulative gain
